# Progress in epigenetic research of breast cancer: a bibliometric analysis since the 2000s

**DOI:** 10.3389/fonc.2025.1619346

**Published:** 2025-09-05

**Authors:** Hua Yang, Yu Fang, Haijuan Wang, Ting Lu, Qihua Chen, Hui Liu

**Affiliations:** ^1^ The First Hospital of Hunan University of Chinese Medicine, Changsha, China; ^2^ Department of Pathology, The First Hospital of Hunan University of Chinese Medicine, Changsha, China; ^3^ Medical College, Hunan University of Chinese Medicine, Changsha, China; ^4^ Medical Department, The First Hospital of Hunan University of Chinese Medicine, Changsha, China

**Keywords:** bibliometric study, epigenetics, breast cancer, visualization, bibliometric, citespace, VOSviewer

## Abstract

**Background:**

Breast cancer continues to be a leading cause of cancer-related deaths among women worldwide. Despite advancements in diagnostics and therapies, challenges such as metastasis, recurrence, and resistance remain prevalent. Recently, research has shifted from traditional genomic analyses to the study of epigenetic regulation, which includes DNA methylation, histone modifications, and non-coding RNAs. Given the rapid expansion of literature in this field, a systematic overview of its evolution and emerging trends is necessary.

**Methods:**

We performed a comprehensive bibliometric analysis of 5,271 articles on breast cancer epigenetics, sourced from the Web of Science Core Collection, covering the years 2000 to 2024. Utilizing tools like CiteSpace and VOSviewer, with support from RStudio, Pajek, and HisCite, we analyzed co-citation networks, keyword co-occurrence, and burst detection. This analysis included visualizations of collaboration among authors, institutions, and countries. Metrics such as modularity, silhouette scores, and betweenness centrality were used to ensure analytical rigor and to identify thematic evolution and emerging research frontiers.

**Results:**

From 2000 to 2018, the number of annual publications increased steadily, with citation peaks occurring in 2021. The United States led in research output and influence, followed by China. Leading institutions included Johns Hopkins University and the University of Texas MD Anderson Cancer Center. Keyword and co-citation analyses revealed four research phases: (1) early studies focused on promoter hypermethylation of tumor suppressor genes like RASSF1A; (2) an in-depth investigation of molecular mechanisms, including epithelial-mesenchymal transition and chromatin remodeling; (3) translational research involving HDAC inhibitors and DNA methylation biomarkers; and (4) recent advancements in multi-omics integration, synthetic lethality, and the study of epigenetics in the tumor microenvironment. Emerging research directions include the targeted removal of epigenetic memory, metabolism-epigenetics networks, and single-cell epigenomic profiling.

**Conclusion:**

This bibliometric study outlines the trajectory of research in breast cancer epigenetics, highlighting its evolution from basic methylation studies to advanced therapeutic exploration. Future research should focus on targeting epigenetic memory to combat drug resistance and recurrence, developing synthetic lethality strategies, and employing single-cell technologies for dynamic epigenetic mapping. These findings provide a strategic roadmap for researchers and policymakers navigating the evolving landscape of breast cancer epigenetics.

## 1 Background

Breast cancer is the most prevalent malignancy affecting women globally. In 2022, there were approximately 2.3 million new cases and 670,000 deaths, as reported (1). There is significant heterogeneity in clinical outcomes and therapeutic responses across different molecular subtypes. Despite advances in early detection and targeted therapies, challenges such as drug resistance, metastasis, and recurrence persist, indicating that breast cancer is the leading cause of cancer-related deaths among women. Although traditional genomic approaches can identify mutations like BRCA1/2 and PIK3CA, they fail to fully account for the dynamic adaptability of tumor cells or the role of non-genetic factors on disease progression (2). This knowledge gap has redirected research focus toward epigenetic regulation—a reversible and heritable mechanism that modulates gene expression without altering DNA sequences—positioning it at the forefront of oncological research.

Epigenetic mechanisms—such as DNA methylation, histone modifications, chromatin remodeling, and non-coding RNAs—play a crucial role in breast carcinogenesis (3). For instance, hypermethylation of promoter regions in tumor suppressor genes such as RASSF1A and BRCA1 occurs in 40%–60% of breast cancers cases, correlating with advanced stages and poor prognosis (4). Conversely, global hypomethylation in triple-negative breast cancer (TNBC) leads to genomic instability and facilitates immune evasion. Histone deacetylases (HDACs) and methyltransferases, such as EZH2, frequently exhibit dysregulation, reshaping the tumor microenvironment (TME) and maintaining stemness (5). The clinical relevance of these findings is highlighted by FDA-approved epigenetic therapies, including HDAC inhibitors like Entinostat and DNMT inhibitors (6).

Over the past two decades, the field has experienced exponential growth, with pioneering studies such as The Cancer Genome Atlas (TCGA) pan-cancer analyses linking epigenetic subtypes to therapeutic vulnerabilities (7). However, the rapid surge in research outputs has fragmented the research landscape, obscuring evolutionary trends, collaborative networks, and emerging frontiers. Bibliometric analysis, a quantitative method for evaluating publication patterns, authorship contributions, and keyword dynamics, provides a systematic approach to map this complex domain. Since its introduction by Pritchard in 1969 (8), bibliometrics has evolved into various computational tools like VOSviewer and CiteSpace, which analyze publications and co-cited references to identify research shifts and trends.

To date, there has been no comprehensive bibliometric assessment of breast cancer epigenetics, which limits the strategic prioritization of research efforts. This study addresses this gap by analyzing 5,271 publications from 2000 to 2024. We utilize co-citation networks, keyword clustering, and burst detection to: (1) describe the temporal evolution of the field; (2) identify influential contributors and institutions; (3) characterize thematic shifts from locus-specific methylation to pan-cancer epigenetic reprogramming; and (4) forecast future directions including synthetic lethality strategies and single-cell epigenomic profiling. By integrating multidimensional bibliographic data, this work provides a roadmap for researchers, clinicians, and policymakers to understand the transformative potential of epigenetics in breast oncology.

## 2 Methods

### 2.1 Data source and search strategy

**Figure 1:**
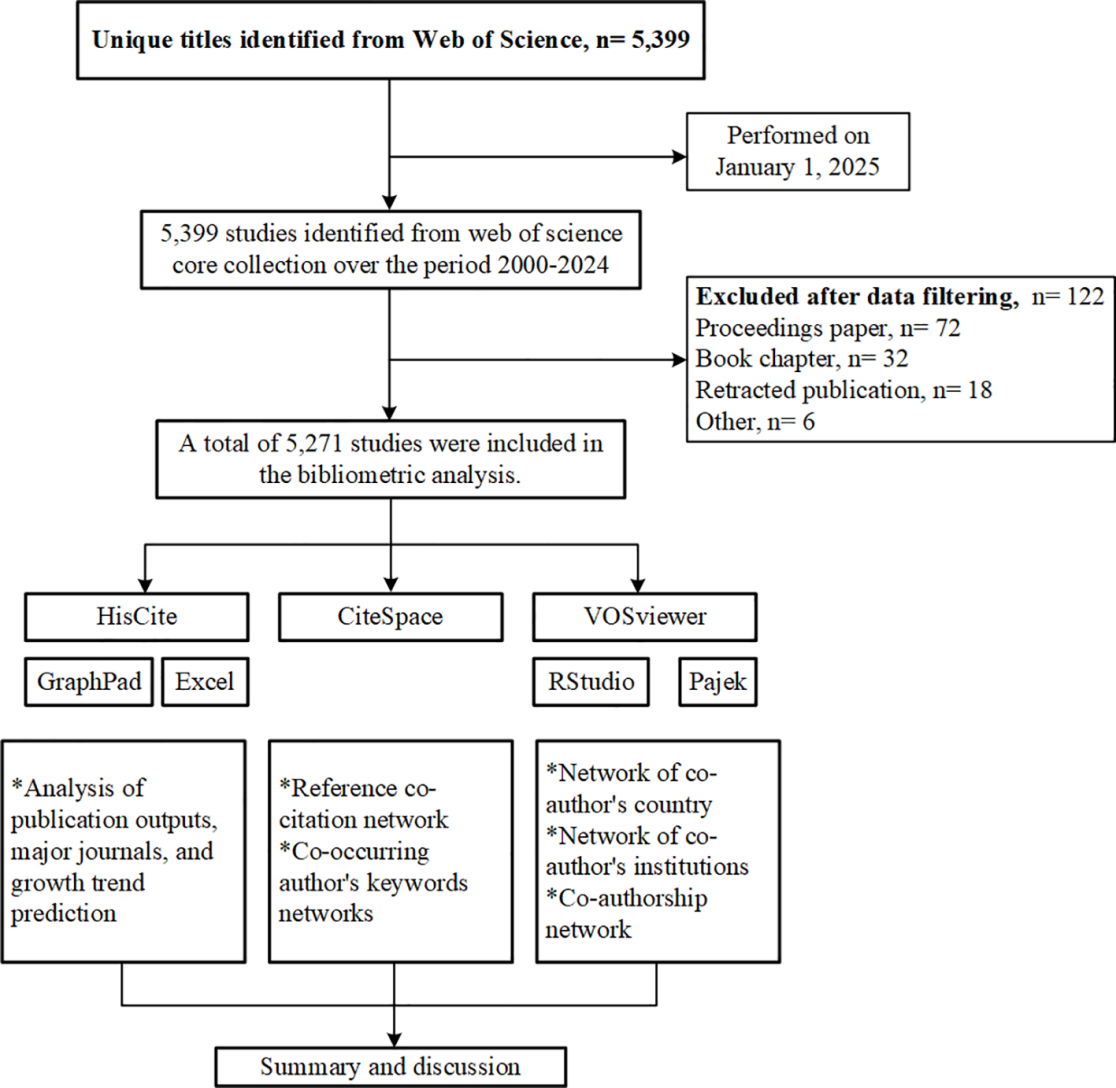
Flow chart of the bibliometric study.

The bibliometric analysis was conducted via the Web of Science Core Collection (WoSCC), a well-recognized and frequently used database for bibliometric research, due to its comprehensive overview of essential data such as publications, citations, authors, references, and keywords. The search strategy utilized was as follows: TS=(“Epigenet*” OR “Epigenesis, Genetic” OR “Epigenetic Processes”) AND TS=(“Breast Cancer” OR “Breast Neoplasm*” OR “Mammary Cancer” OR “Breast Tumor*” OR “Breast Malignan*” OR “Breast Lesion*”) AND DT=(Article). Literature published between January 1, 2000, and December 31, 2024, was retrieved, and the data was confined to the Science Citation Index-Expanded (SCIE) database. Additionally, documents categorized as “retracted paper” “proceedings paper,” and “book chapter” were excluded. The retrieval process was conducted on a single day (January 1, 2025) to minimize potential confounding bias from daily database updates. To ensure the authenticity and reliability of the research data, two trained investigators independently collected the data, and a third colleague intervened in discussions only when divergent opinions needed resolution. Ultimately, 5,271 documents were obtained after manual reference screening, and full records along with cited references were downloaded in plain text for further analysis. A detailed flowchart of the study procedures is provided in **Figure 1**.

### 2.2 Data analysis and visualization

CiteSpace (version 6.4.R1 Advanced) and VOSviewer (version 1.6.17) were the primary tools used, supplemented by additional data analysis and visualization software, including RStudio (version 2024.09.1), Pajek (version 5.19, 64-bit), HisCite (version 12.03.17), GraphPad Prism (version 10.1.2), and Microsoft Excel 2021, to complete the research. These tools facilitated key bibliometric analyses, such as co-citation analysis of references, co-occurrence analysis of keywords, and collaboration network analysis of authors, institutions, and countries. Co-citation analysis, a bibliometric method that examines the frequency with which two documents are cited together in subsequent publications, is a powerful approach for identifying influential literature and integrating cross-disciplinary research ideas (9). Co-occurrence refers to the simultaneous appearance of two keywords within the same publications. Co-authorship illustrates collaborative contributions between research entities and serves as an indicator of cooperation among authors, institutions, or countries (10). To examine recent research trends, the study period focused on the last five years (2020–2024) and the most recent year (2024), with all analyses repeated for these specific time frames.

CiteSpace, developed by Professor Chaomei Chen, is a primary software tool for analyzing medical research trends (11). In this study, CiteSpace was employed to conduct clustering, timeline, and burst analyses of co-cited references and co-occurring keywords, along with constructing co-citation networks. In order to achieve better graphical presentation, we clicked on “all in one” and “optimize layout” to separate each cluster and assign different colors (based on keywords), then clicked “find clusters” to obtain the individual clusters. The clustering results were evaluated via structural metrics such as modularity, which assesses network modularization, and silhouette scores, which measure the homogeneity and quality of the clusters. The labels and results were meticulously re-examined to determine whether adjustments were necessary. It is widely acknowledged that a Q value of ≥ 0.3 indicates significant modularity in the network, with higher values reflecting enhanced clustering performance (12, 13). Similarly, an S value of ≥ 0.5 denotes reasonable clustering quality, with values approaching 1 indicating improved network homogeneity (14).

A citation burst refers to a sudden and significant increase in the number of citations for a specific publication within a defined period (15). Clusters with numerous nodes exhibiting strong citation bursts may indicate emerging trends in current or future research. Betweenness centrality measures the extent to which a node acts as a bridge on the shortest paths connecting other node pairs, highlighting its role in the network’s structure. The analysis parameters were as follows: (1) the time frame was from 2000 to 2024; (2) the time slicing interval was one year; and (3) the g-index, an extension of Hirsch’s h-index, was used to evaluate the global citation performance of the article set (16). The quantity of exhibited nodes was determined by the g-index. Due to fluctuations in citation volumes over time frames, the k-values for the g-index were 25 for 2000–2024, 50 for 2020–2024, and 100 for 2024; (4) all other parameters were left at their normal settings.

VOSviewer is a sophisticated tool for viewing and mapping diverse network data, and is proficient at processing extensive datasets and generating high-quality network maps (17). This study employed VOSviewer to generate collaboration network maps for countries, institutions, and authors. Before constructing the national collaboration network map, we first assigned the geographical data of the involved countries in RStudio, primarily using the “sp”, “rgdal”, and “tidyverse” packages. Then, we used VOSviewer for visualization. Detailed methods can be found at https://github.com/like-firework/Progress-in-Epigenetic-Research-of-Breast-Cancer. The research of institutional and author partnerships utilized VOSviewer to construct the network, which was further modified with Pajek. Citation data for the papers were evaluated with HisCite software to identify highly cited articles, and bar charts were created via GraphPad. Excel was used for data storage during the investigation.

## 3 Results

### 3.1 Analysis of co‐cited references: publications, most cited papers, and research clusters

#### 3.1.1 Analysis of publications

**Figure 2:**
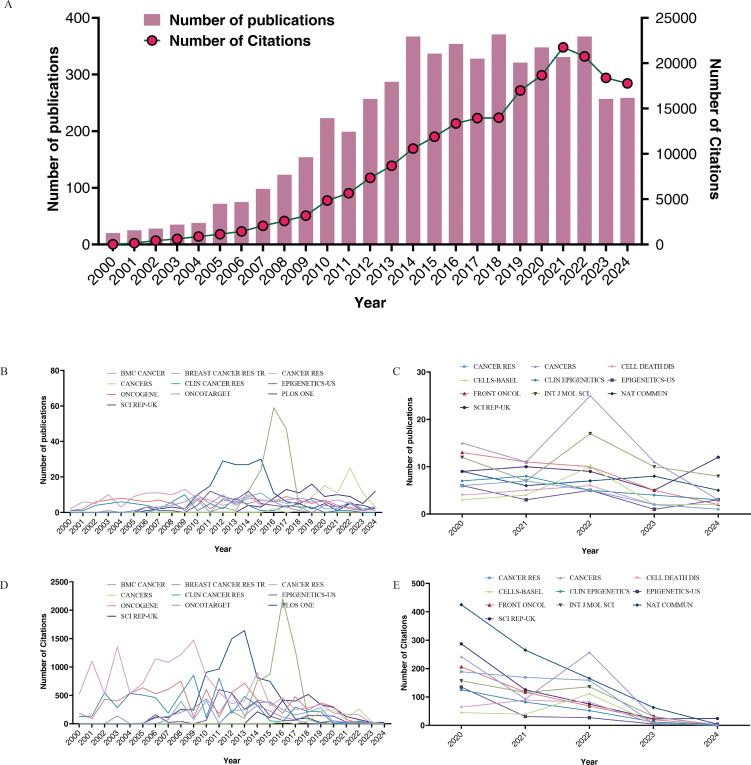
Annual number of publications and citations from 2000 to 2024 **(A)**, top 10 journals by publication volume for the time period 2000-2024 **(B)** and 2020-2024 **(C)**, top 10 cited journals for the time period 2000-2024 **(D)** and 2020-2024 **(E)**.

As of December 31, 2024, a total of 5,271 articles have been published within this field, indicating significant research activity. **Figure 2A** illustrates the annual trends in both publication and citation counts. Our analysis reveals a substantial increase in both metrics. The lowest publication output occurred in 2000 with 20 articles, whereas the peak was in 2018 with 371 articles. The number of citations were lowest in 2000, totaling 17, and reached a maximum in 2021, with 21,749 citations. The sustained high number of published papers over the past decade highlights the popularity and maturation of this research field.

**Table 1** presents the journals with the highest number of published papers and their respective citation frequencies. We identified the top 15 journals based on publications for the periods 2000–2024 and 2020–2024. Notably, *PLOS ONE* has the highest volume of published articles; however, each article in *CANCER RES* receives, on average, 2.1 times more citations than those in *PLOS ONE*, indicating that the papers published in this journal have a higher long-term impact in the field. Journals such as *ONCOTARGET*, *ONCOGENE*, and *SCI REP-UK* also demonstrated a considerable number of publications. Over the past five years, the number of relevant publications in journals such as *CANCERS*, *INT J MOL SCI*, and *SCI REP-UK* has increased. Based on standardized citations (referring to the total number of citations a journal has received divided by the total number of articles it has published), we believe that during the period from 2000 to 2024, articles published in *CANCER RES* and *CLIN CANCER RES* exhibited relatively high impact. In the past five years, the leading journals have been *SEMIN CANCER BIOL* and *NAT COMMUN*. Furthermore, we examined the publication counts and citation trends of the top 10 journals from both 2000–2024 and 2020–2024, as illustrated in **Figures 2B–E**.

**Table 1:** Top 15 journals for publications.

2000 - 2024					2020 - 2024				
Rank	Journal	Publications	Citations	Standardized citations	Rank	Journal	Publications	Citations	Standardized citations
1	PLOS ONE	187	8015	42.86	1	CANCERS	65	623	9.58
2	CANCER RES	164	14886	90.77	2	INT J MOL SCI	54	441	8.17
3	ONCOTARGET	149	5507	36.96	3	SCI REP-UK	45	534	11.87
4	ONCOGENE	129	8724	67.63	4	FRONT ONCOL	41	412	10.05
5	SCI REP-UK	100	2520	25.20	5	NAT COMMUN	35	922	26.34
6	BMC CANCER	93	3179	34.18	6	CLIN EPIGENETICS	27	272	10.07
7	EPIGENETICS-US	92	2838	30.85	7	CANCER RES	21	524	24.95
8	BREAST CANCER RES TR	85	2996	35.25	8	CELLS-BASEL	21	203	9.67
9	CLIN CANCER RES	78	6903	88.50	9	EPIGENETICS-US	18	202	11.22
10	CANCERS	77	932	12.10	10	CELL DEATH DIS	18	242	13.44
11	INT J CANCER	75	5136	68.48	11	FRONT GENET	18	104	5.78
12	INT J MOL SCI	74	1046	14.14	12	SEMIN CANCER BIOL	18	696	38.67
13	NAT COMMUN	72	5025	69.79	13	PLOS ONE	17	115	6.76
14	CLIN EPIGENETICS	71	1730	24.37	14	FRONT CELL DEV BIOL	16	238	14.88
15	BREAST CANCER RES	69	3517	50.97	15	ONCOGENE	16	400	25.00

“Standardized citations” refers to the total number of citations a journal has received divided by the total number of articles it has published.

#### 3.1.2 The most cited papers

We have identified the 10 most referenced works in both the literature and the network from 2000 to 2024, as detailed in **Tables 2** and **3**. The most cited paper is by Koboldt et al., with 9,254 citations in the literature and 270 in the network. This study provides a comprehensive molecular characterization of breast cancer subtypes, emphasizing mutations in TP53, PIK3CA, and GATA3 (18). The research of Horvath et al. follows, with 4,045 citations, introducing a universal age predictor on the basis of DNA methylation and identifying age acceleration in cancer tissues (19). The third most cited study is that of Irizarry et al., which was cited 1,670 times and shows that DNA methylation changes in colon cancer occur mainly at CpG island shores, affecting gene expression (20). Additionally, in the network, Burbee et al.’s and Fackler et al.’s studies, with 64 and 56 citations respectively, explored the role of RASSF1A and other genes in breast cancer epigenetics (4, 21).

**Table 2:** The top 10 most frequently cited papers in the literature between 2000 and 2024.

Number of citations in the network	Number of citations in the literature	Average annual citations	Self-citation rate(%)	Title	Year	Vol	Page	Doi	Source
270	9242	660.14	6.73	Comprehensive molecular portraits of human breast tumours	2012	490	7418	10.1038/nature11412	Nature
0	4045	311.15	5.88	DNA methylation age of human tissues and cell types	2013	14	10	10.1186/gb-2013-14-10-r115	Genome Biol.
56	1670	98.24	2.34	The human colon cancer methylome shows similar hypo- and hypermethylation at conserved tissue-specific CpG island shores	2009	41	2	10.1038/ng.298	Nat. Genet.
0	1307	93.36	3.14	methylKit: a comprehensive R package for the analysis of genome-wide DNA methylation profiles	2012	13	10	10.1186/gb-2012-13-10-R87	Genome Biol.
0	1174	69.06	3.32	An Epigenetic Switch Involving NF-κB, Lin28, Let-7 MicroRNA, and IL6 Links Inflammation to Cell Transformation	2009	139	4	10.1016/j.cell.2009.10.014	Cell
15	998	124.75	2.00	Identification of the tumour transition states occurring during EMT	2018	556	7702	10.1038/s41586-018-0040-3	Nature
3	915	83.18	3.50	Microenvironment-induced PTEN loss by exosomal microRNA primes brain metastasis outgrowth	2015	527	7576	10.1038/nature15376	Nature
7	869	51.12	0.69	Heterogeneity in Cancer: Cancer Stem Cells versus Clonal Evolution	2009	138	5	10.1016/j.cell.2009.08.017	Cell
34	857	47.61	8.17	A microRNA DNA methylation signature for human cancer metastasis	2008	105	36	10.1073/pnas.0803055105	Proc. Natl. Acad. Sci. U.S.A.
0	805	80.50	0.50	HDACs and HDAC Inhibitors in Cancer Development and Therapy	2016	6	10	10.1101/cshperspect.a026831	Cold Spring Harb. Perspect. Med.

**Table 3:** The top 10 most frequently cited papers in the network between 2000 and 2024.

Number of citations in the network	Number of citations in the literature	Average annual citations	Self-citation rate(%)	Title	Year	Vol	Page	Doi	Source
270	9254	661.00	6.72	Comprehensive molecular portraits of human breast tumours	2012	490	1374	10.1038/nature11412	Nature
64	648	25.92	20.68	Epigenetic inactivation of RASSF14 in lung and breast cancers and malignant phenotype suppression	2001	93	26	10.1093/jnci/93.9.691	J. Natl. Cancer Inst.
56	210	9.13	9.05	DNA methylation of RASSF1A, HIN-1, RAR-beta, Cyclin D2 and Twist in *in situ* and invasive lobular breast carcinoma	2003	107	118	10.1002/ijc.11508	Int. J. Cancer
56	1672	98.35	2.33	The human colon cancer methylome shows similar hypo- and hypermethylation at conserved tissue-specific CpG island shores	2009	41	594	10.1038/ng.298	Nat. Genet.
55	207	11.50	3.38	Frequent epigenetic inactivation of Wnt antagonist genes in breast cancer	2008	98	459	10.1038/sj.bjc.6604259	Br. J. Cancer
52	213	10.65	13.62	Aberrant methylation of the Wnt antagonist SFRP1 in breast cancer is associated with unfavourable prognosis	2006	25	268	10.1038/sj.onc.1209386	Oncogene
50	258	11.22	8.53	DNA methylation in serum of breast cancer patients:: An independent prognostic marker	2003	63	107	NA	Cancer Res.
46	226	10.27	13.27	Quantitative multiplex methylation-specific PCR assay for the detection of promoter hypermethylation in multiple genes in breast cancer	2004	64	138	10.1158/0008-5472.CAN-03-3341	Cancer Res.
45	467	25.94	6.00	Repression of E-cadherin by the polycomb group protein EZH2 in cancer	2008	27	578	10.1038/onc.2008.333	Oncogene
44	376	26.86	6.65	G9a interacts with Snail and is critical for Snail-mediated E-cadherin repression in human breast cancer	2012	122	1247	10.1172/JCI57349	J. Clin. Invest.

In the past five years, many studies, including Becker et al.’s 2020 study, have focused on cancer-associated fibroblasts and their epigenetic and metabolic roles in breast cancer under hypoxic conditions (22). Research by Corr et al.’s 2020 research demonstrated how myocardial infarction might accelerate the progression of breast cancer by reprogramming immune-suppressive monocytes through epigenetic mechanisms (23). In 2021, Chen et al. systematically analyzed RNA adenosine modifications in colorectal cancer and developed a WM_Score model for predicting patient survival and TME features (24). Collectively, these works underscore the crucial role of epigenetic regulation in cancer progression and TME dynamics.

Furthermore, we assessed the impact of publications from 2000–2024 and 2020–2024 by evaluating citation bursts (Additional file 2: **Supplementary Table S1**). The timeline is depicted with a segmented blue line indicating each year, while a red line denotes the duration of citation bursts. Notably, the strongest and most recent citation bursts are from Sung et al.’s “Global Cancer Statistics 2020,” (25) Waks and Winer’s “Breast Cancer Treatment: A Review,” (26) and Loibl et al.’s “Breast Cancer.” (27).

#### 3.1.3 Cluster of research

We constructed cluster-based co-citation networks for the periods 2000–2024, 2020–2024, and 2024. All three networks demonstrated well-structured and reliable configurations, with the 2000–2024 network attaining a modularity (Q) of 0.7311 and a silhouette score (S) of 0.8866, the 2020–2024 network achieving Q = 0.691 and S = 0.8481, and the 2024 network reaching Q = 0.6597 and S = 0.8634. Each reference was depicted as a single node, with node size reflecting co-citation frequency. The citation rings were color-coded in chronological order, and their thickness was proportional to the number of citations within the corresponding time frame. The arrows indicate dependency links among clusters. Detailed descriptions of the major clusters of co-cited references can be found in Additional File 3: **Supplementary Table S2**, and the visualization of each network is shown in Additional File 1: **Supplementary Figures S1** and **S2**. Additionally, landscape visualization depicts the start, duration, and cessation of each cluster, where peak heights represent cluster activity levels.

**Figure 3:**
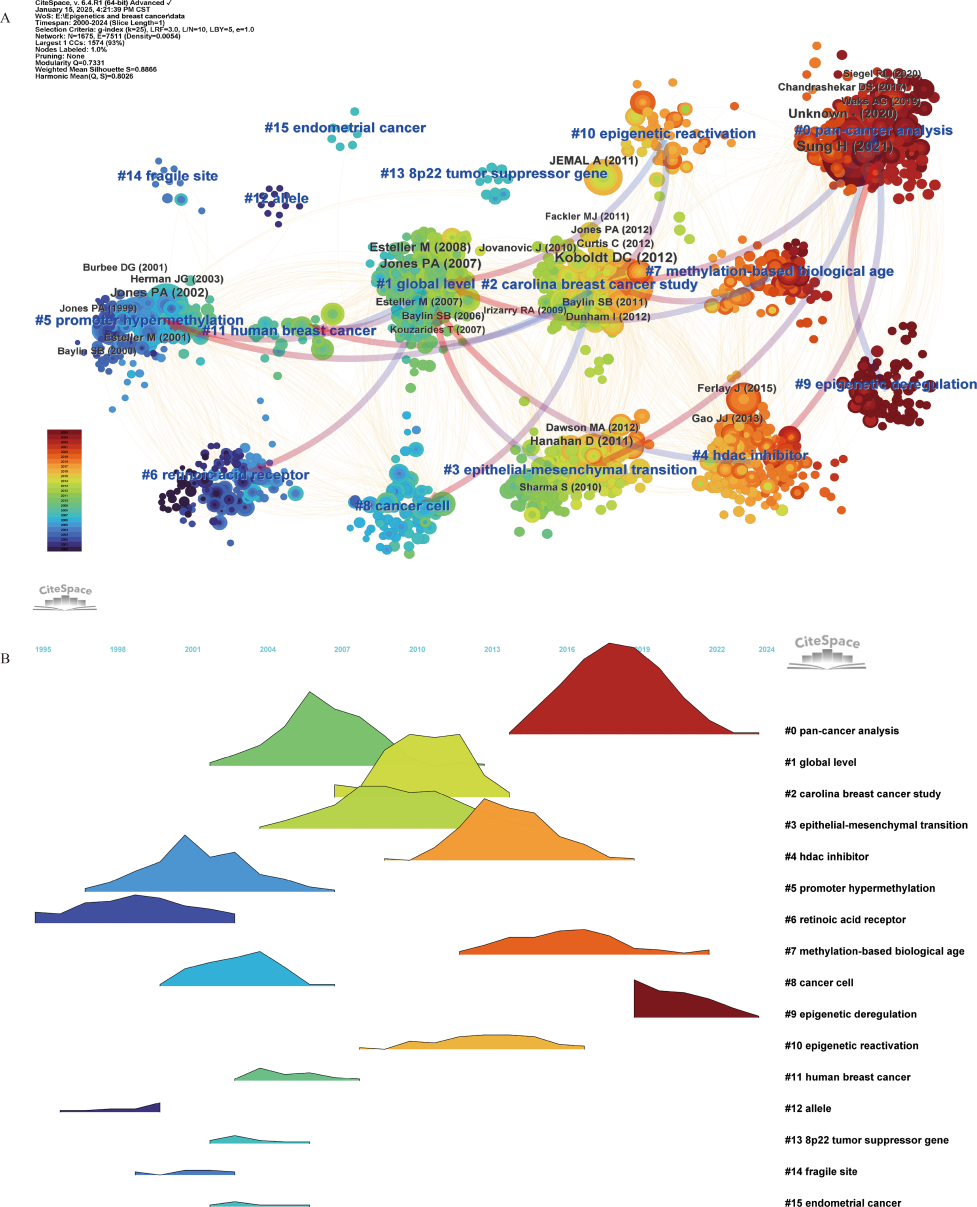
Co-citation reference (2000–2024) network and clustering visualization of hotspots, and citation relationships between 16 clusters **(A)**. Landscape Visualization of clusters from 2000 to 2024 **(B)**. Each node represents a co-cited reference, and the size of the node is proportional to its co-citation frequency. The tree rings around each node indicate the citation burst period. The gradient from blue to red corresponds to the time span from 2000 to 2024, with dark blue representing 2000 and dark red representing 2024. Blue labels denote the cluster number and name, where cluster #0 is the smallest and cluster #15 is the largest. The arrows indicate dependency links among clusters.

In the co-citation network from 2000 to 2024, we identified 16 distinct clusters. Clusters were numerically designated by size, with the largest as #0 and the smallest as #15. Each cluster was characterized by qualitative measures, including label, size, silhouette score, and the average year of co-cited references. Landscape visualization succinctly illustrates the evolution of research themes in this field since 2000 (**Figure 3**). The silhouette scores of the identified clusters are all greater than 0.8, indicating that the members within each cluster exhibit good homogeneity. Based on the analysis of these clusters, we suggest that one of the earliest identified epigenetic alterations in breast cancer is the loss of gene expression resulting from promoter hypermethylation.

This hypothesis is substantiated by five early clusters: #12 “allele”, #6 “retinoic acid receptor”, #14 “fragile site”, #5 “promoter hypermethylation”, and #13 “8p22 tumor suppressor gene”. Research subsequently deepen, with studies like #1 “global level” and #2 “Carolina breast cancer study” focusing on metastasis and cancer progression, particularly #3 epithelial-mesenchymal transition. Over the past decade, emphasis has been placed on epigenetic regulatory mechanisms, including #10 “epigenetic reactivation” and #9 “epigenetic deregulation”; predictive measures like #7 “methylation-based biological age”; and treatment modalities exemplified by #4 “HDAC inhibitor”. Notably, pan-cancer analysis has gained prominence in recent years, exemplified by cluster #0 pan-cancer. Detailed information for each cluster can be found in Additional File 3: **Supplementary Table S2** A.

**Figure 4:**
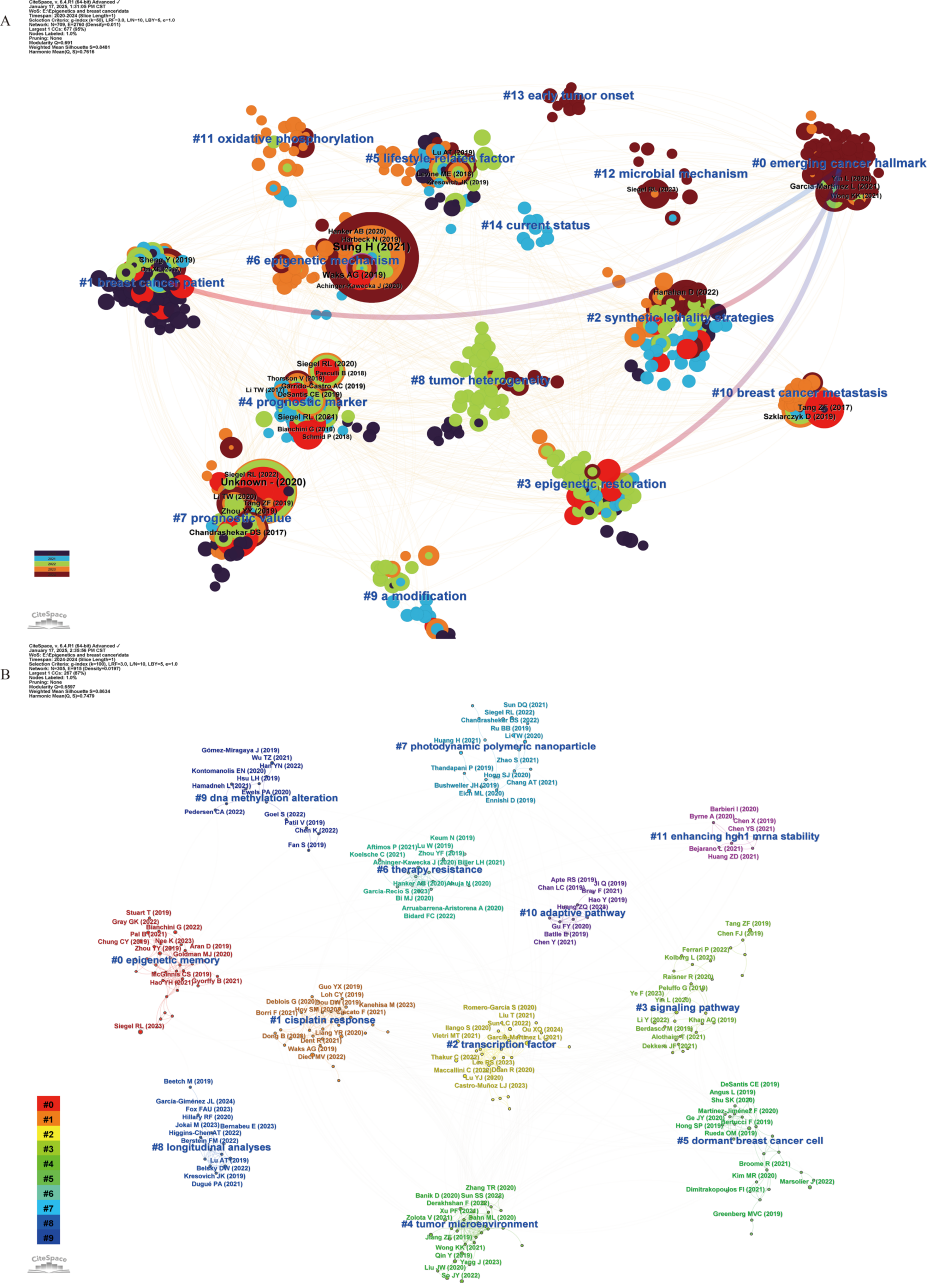
Co-citation network of references with corresponding clusters obtained with CiteSpace for the time period 2020-2024 **(A)** and 2024 **(B)**. Each node represents a co-cited reference, and the size of the node is proportional to its co-citation frequency. The tree rings around each node indicate the citation burst period. Blue labels indicate the cluster number and name, where Cluster #0 is the smallest and Cluster #15 is the largest. In Panel **(A)**, the gradient from red to blue represents the time span from 2020 to 2024, with dark red indicating 2020 and dark blue indicating 2024. Arrows denote the dependency relationships among clusters. In Panel **(B)**, the gradient from purple to red corresponds to clusters ranging from smallest to largest, with purple representing the smallest cluster (#11) and red indicating the largest cluster (#15).

In addition, we analyzed the co-cited references published over the past 5 years (2020–2024) and the most recent year (2024) (**Figure 4**) to understand the latest research trends, detailed information can be found in Additional File 3: **Supplementary Table S2**, sections B and C. Consistent with our previous analyses, scholars over the past five years have primarily focused on exploring epigenetic regulatory mechanisms and therapeutic strategies. In the 2020–2024 network, biomarker screening has emerged as a major research focus, as reflected in cluster #0 “emerging cancer hallmark” and cluster #4 “prognostic marker”. Moreover, #8 “tumor heterogeneity”, #11 “oxidative phosphorylation”, and #12 “microbial mechanism” represent important directions in pathogenesis research. The network highlights #2 “synthetic lethality strategies” and #3 “epigenetic restoration” as emerging epigenetic intervention strategies. In the 2024 network, #0 “epigenetic memory”, #5 “dormant breast cancer cell”, and chemotherapy resistance in breast cancer—illustrated by #1 “cisplatin response” and #6 “therapy resistance”—are considered major research directions.

### 3.2 Analysis of co‐occurrence of keywords

The primary goal of constructing a co-occurrence network of keywords and conducting burst analysis is to offer a comprehensive overview of the current research landscape and predict the evolution of research hotspots over time. Within this network, each node represents a frequently co-occurring keyword, with node size reflecting the frequency of occurrence and the color of the annual ring indicating the time of appearance.

**Figure 5:**
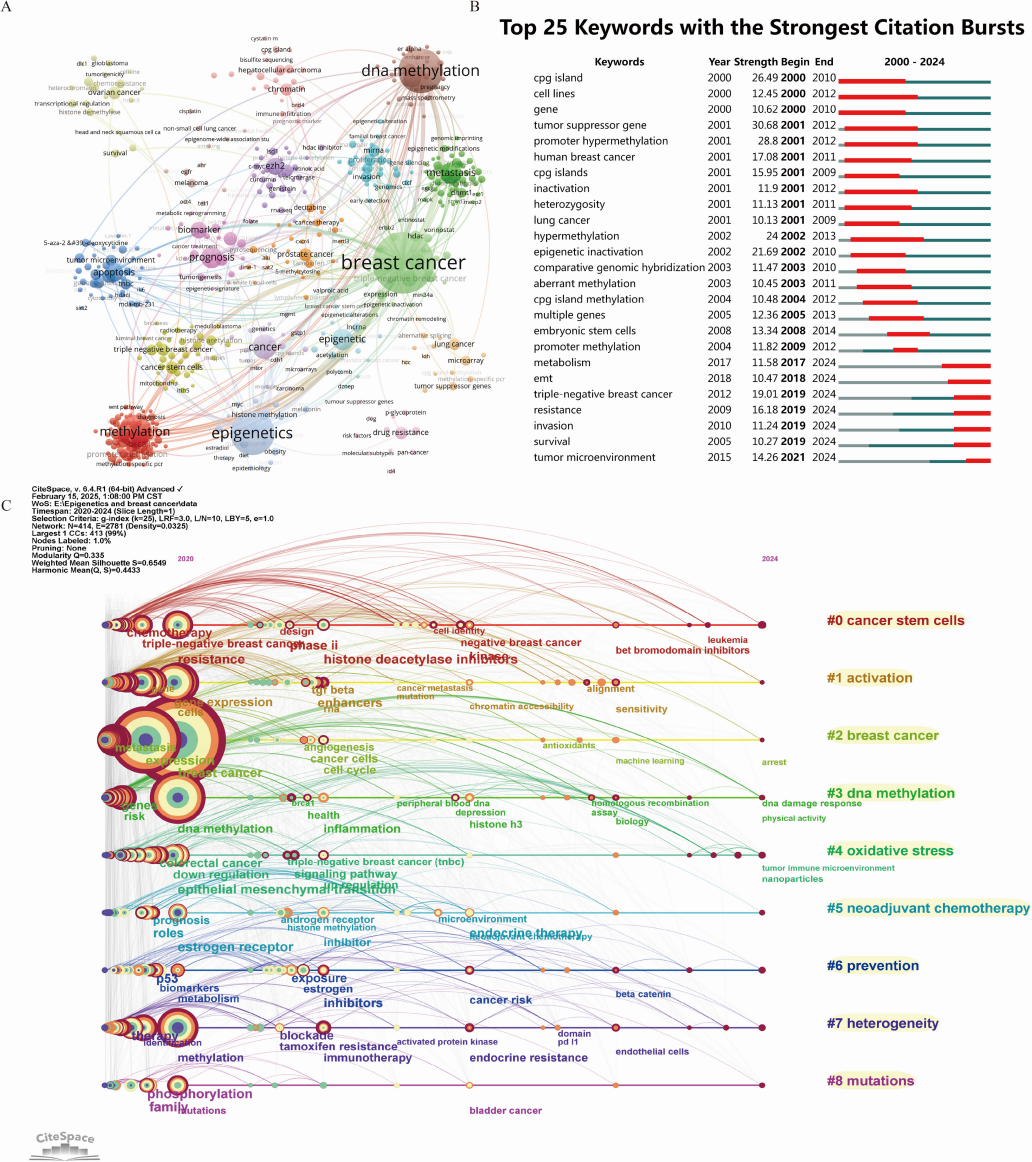
Visualization of the co-occurring network of keywords from 2000 to 2024 **(A)**, citation burst analysis of keywords between 2000 and 2024 **(B)**, and timeline visualization of the network **(C)** for the time period 2020-2024. In Panel **(A)**, each node represents a keyword. The larger the node, the greater the number of associated publications. Keywords belonging to the same category are shown in the same color. In panel B, red indicates the duration of a burst, indicating that keywords are frequently quoted, while green indicates keywords that are not frequently quoted. In Panel **(C)**, each node represents the time when a keyword first appeared. The larger the node, the more frequently the keyword appeared. The rings surrounding the nodes indicate the duration over which the keyword continued to appear. Purple lines represent the earliest occurrence (2020), while red lines represent the most recent (2024). The cluster labels are generated by the CiteSpace software based on the timeline displayed on the right side of the figure.

Between 2000 and 2024, key terms in the network include breast cancer, growth, DNA methylation, metastasis, proliferation, epigenetics, gene expression, prognosis, BRCA1, tumor suppressor, and epigenetic regulation. The connections among these keywords are depicted via VOSviewer in **Figure 5A**. The three keywords exhibiting the highest burst strengths are tumor suppressor genes, promoter hypermethylation, and CpG islands, with their evolutionary trajectories shown in **Figure 5B**.

Keyword changes over the past five years serve as crucial indicators of emerging research hotspots. Therefore, a clustering analysis of keywords from 2020 to 2024 was performed, resulting in eight clusters (Q=0.335, S=0.6549, **Figure 5C**): #0 cancer stem cells, #1 activation, #2 breast cancer, #3 DNA methylation, #4 oxidative stress, #5 neoadjuvant chemotherapy, #6 prevention, #7 heterogeneity, and #8 mutations. Detailed information on each cluster is available in Additional File 4: **Supplementary Table S3** A. In the burst term analysis, histone H3, microenvironment, and liquid biopsy emerged as the latest hotspot terms, as detailed in Additional File 4: **Supplementary Table S3** B.

### 3.3 Analysis of the cooperation network across authors, institutions and countries

**Figure 6:**
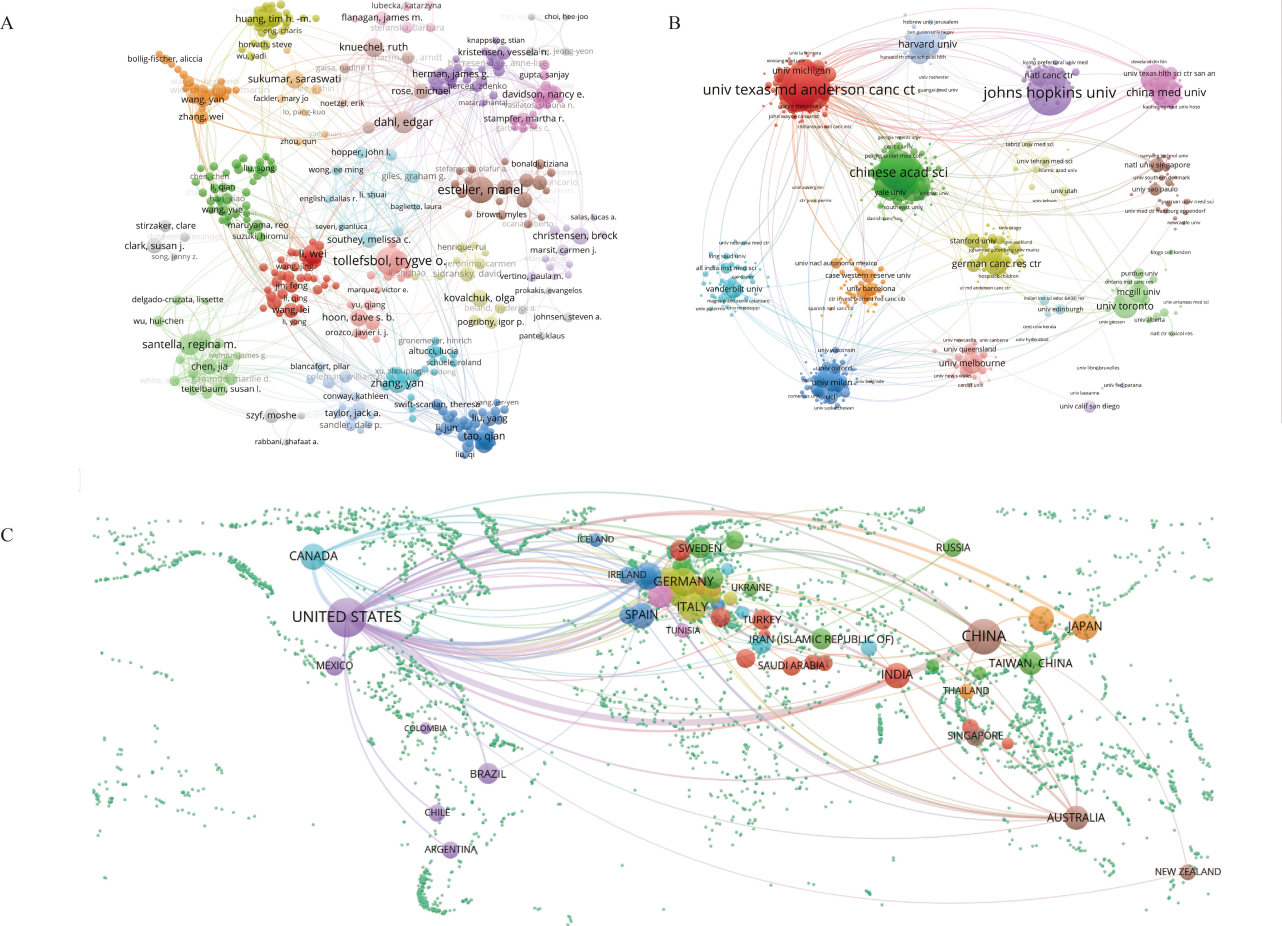
Visualization of the co-occurring network of authors **(A)**, institutions **(B)**, and countries **(C)** from 2000 to 2024. In Panels **(A-C)**, the nodes represent authors, institutions, and countries, respectively. Larger nodes indicate a greater number of associated publications. Elements belonging to the same category are displayed in the same color.

Using VOSviewer, we constructed co-occurrence networks for authors, institutions, and countries (**Figure 6**). Within the author co-occurrence network, 22 major collaboration networks were identified. By employing the average citation score (citations per paper) as the primary metric of impact, Horvath Steve, Yao Jun, Hung Mien-Chie, Zhang Lin, and Brown Myles were identified as the most influential authors, with Yao Jun and Zhang Lin in the same cluster. Among the 16 major institutional collaboration networks, Johns Hopkins University (101 publications), University of Texas MD Anderson Cancer Center (98 publications), NCI (93 publications), Chinese Academy of Sciences (85 publications), and Shanghai Jiao Tong University (77 publications) had the highest number of publications. Johns Hopkins University (9,365 citations), Harvard University (7,892 citations), and the University of Texas MD Anderson Cancer Center (7,648 citations) received the most citations. Furthermore, the University of Texas MD Anderson Cancer Center presented the most interinstitutional connections. Additionally, the countries with the highest number of publications were the United States (2,072 publications), China (1,258 publications), the UK (345 publications), Germany (334 publications), Italy (292 publications), and Canada (240 publications). International collaborations predominantly occurred between neighboring countries, with the United States facilitating the most connections. In addition, detailed information on publications by authors, institutions, and countries can be found in Additional File 4: **Supplementary Tables S4**-**S6**, respectively.

## 4 Discussion

### 4.1 Summary of the main findings

The bibliometric analysis revealed a continuing increase in annual publications from 2000 (*n* = 20) to 2018 (*n* = 371), with citation counts peaking at 21,749 in 2021. This trend underscores the progressive development of epigenetic research on breast cancer and advances in related analysis techniques. Geographically, the United States published the most papers, contributing 2,072 articles (39.3% of the total), followed by China with 1,258 articles (23.9% of the total). When it comes to the perspective of research impact, countries such as Pakistan, Finland, and Russia achieved higher citation counts despite having published fewer papers. At the institutional level, Johns Hopkins University leads in paper production (101 papers), whereas the University of Texas MD Anderson Cancer Center stands out for its impact, with 7,648 citations and the highest interinstitutional connectivity (degree centrality = 0.43). Notably, Chinese institutions such as the Chinese Academy of Sciences and Shanghai Jiao Tong University are emerging. In terms of journal publications, *PLOS ONE* and *CANCER RESEARCH* have the most publications, but *CANCER RESEARCH* is cited 2.1 times more often per article than *PLOS ONE*. Additionally, Horvath Steve, Yao Jun, and Hung Mien-Chie are notable for publishing the most papers in this field. They have made significant contributions to the study of epigenetic age acceleration and breast cancer risk, as well as to the regulation of key epigenetic factors (28–30).

### 4.2 Evolution of epigenetic research topics in breast cancer and key articles

Through clustering and burst analyses of co-cited references, combined with a landscape visualization of clusters from 2000 to 2024 (**Figure 3**), we have identified four distinct phases in the evolution of breast cancer epigenetics research hotspots: the initial exploration phase, the systematic elucidation of molecular mechanisms, the clinical translation phase, and the emergence of novel insights into breast cancer epigenetics.

#### 4.2.1 The budding phase

During this period, research focused on the promoter hypermethylation of tumor suppressor genes, particularly in the chromosome 8p22 region. Key clusters include #5 promoter hypermethylation (Year = 2001), #12 allele (Year = 1998), #13 8p22 tumor suppressor gene (Year = 2001), and #14 fragile site (Year = 2001). A seminal paper by Burbee et al. (21) first demonstrated the hypermethylation of the RASSF1A gene promoter in breast cancer, highlighting epigenetic silencing as a mechanism for oncogene inactivation, and paving the way for further studies on promoter methylation in other genes (21). Müller et al. found that serum RASSF1A and APC DNA methylation are strongly associated with poor prognosis in breast cancer patients, serving as crucial indicators for patient prognosis assessment (31). Consistent with the findings of Fackler et al., methylation differences in the promoters of the RASSF1A, Cyclin D2, RAR-beta, and Hin-1 genes were detected between lobular carcinoma *in situ* and invasive lobular carcinoma of the breast (4). In 2018, a study in India also confirmed that hypermethylation of RASSF1A is correlated with a poor prognosis in patients with breast cancer (32). Additional corroboration was found for the correlation between promoter hypermethylation and breast cancer in genes such as TMS1, ACS, DSC3, and BRCA1 (33–35).

#### 4.2.2 Emphasize an in-depth investigation of the molecular mechanisms

The focus during this phase shifted from individual gene methylation changes to comprehensive, systematic elucidation (cluster #1, global level, Year = 2006). Methodological advances have greatly driven progress. In 2004, Fackler et al. introduced a high-sensitivity quantitative multiplex methylation-specific PCR method, allowing the detection of promoter methylation levels across multiple genes with less tissue (36). This method was subsequently used for quantitative gene methylation analysis in breast tissues (37–39). The advent of sequencing technologies such as GS 20 further facilitated the systematic interpretation of breast cancer epigenetics. Gary and colleagues utilized ChIP-seq technology to analyze methylation profiles in breast cancer cell lines and primary human mammary epithelial cells, revealing the unique interplay of DNA methylation with histone modifications the H3K9me3 and H3K27me3 modifications (40). In 2012, Akalin et al. released the methylKit R package to help researchers quickly identify statistically significant DNA methylation sites or regions, easing data analysis, and enhancing research efficiency (41).

At this phase, the Carolina Breast Cancer Study (cluster #2, Year = 2010) had a significant impact as a long-term, population-based research project conducted by the University of North Carolina at Chapel Hill, substantially influencing breast cancer epidemiology, molecular biology, and genetic analysis (42–44). Furthermore, research has expanded from individual gene changes to mechanisms involved in EMT (cluster #3, Year = 2009), such as the discovery by Neha et al. that Sox4 regulates EMT via the epigenetic modifier Ezh2, promoting breast cancer (45).

#### 4.2.3 Epigenetic therapy

Clinical application is the ultimate purpose and value of academic research, serving as the culmination of theoretical endeavors. This study revealed that the primary direction to improve breast cancer prognosis through targeted epigenetics is epigenetic reactivation (#10, Year = 2013), which involves the reactivation of silenced tumor suppressor genes. Among these, HDAC inhibitors (cluster #4, Year = 2013) are representative regulatory drugs that indirectly induce histone acetylation by disrupting HDAC activity, leading to the re-expression of regulatory genes in cancer cells and reversing malignant phenotypes (46). To date, more than 60 clinical studies have been registered in ClinicalTrials.gov for breast cancer treatments (47), including Vorinostat, Entinostat, and Panobinostat (48–50). However, due to their toxicity and risk of overdose, HDAC inhibitors are often combined with drugs like Tamoxifen and Paclitaxel, targeting multiple oncogenic signaling pathways to overcome drug resistance in advanced breast cancer (51–53). DNA methyltransferase inhibitors, such as azacitidine and decitabine, are also a class of epigenetic therapeutic drugs. During DNA replication, these agents bind to DNA methyltransferases, inhibiting gene methylation and subsequently reactivating silenced tumor suppressor genes. Paradoxically, recent research indicates that treatment with these inhibitors may also result in the *de novo* hypermethylation of specific CpG sites (54). This novel mechanism could influence both their therapeutic effects and side effects. Clinically, the U.S. Food and Drug Administration has approved azacitidine and decitabine for the treatment of myelodysplastic syndromes and certain leukemias (55). Although their efficacy as monotherapies for breast cancer remains limited, they have a certain inhibitory effect on tumor invasion and metastasis, particularly in triple-negative breast cancer (56). Recently, an epigenetic therapeutic drug targeting the histone methyltransferase EZH2 has also shown certain potential in the treatment of breast cancer (57).

#### 4.2.4 New insights into epigenetics of breast cancer

Thanks to advancements in whole-genome projects and bioinformatics analysis methods, researchers can integrate multi-omics data (cluster #0, pan-cancer analysis, Year = 2018) to analyze subtype-specific epigenetic characteristics of breast cancer and the regulatory role of the TME (cluster #9, epigenetic deregulation, Year = 2020). Llorente et al. combined proteomics, transcriptomics, epigenomics, chromatin accessibility, and functional analysis, finding that MAF interacts directly with estrogen receptor α, forming a unique chromatin landscape conducive to metastasis. They also discovered that the histone demethylase KDM1A promotes epigenomic remodeling, further impacting breast cancer metastasis (58). In this cluster, Trnkova et al.’s analysis of epigenetic dysregulation in the TME (59), and Zhou et al.’s exploration of the epigenetic regulation of TNBC (60) have significant influence. Moreover, a new direction, methylation-based biological age (cluster #7, Year = 2016), has emerged, which uses mathematical modeling to construct an epigenetic clock for assessing cellular aging and cancer prognosis, as seen in Kresovich et al.’s predictive model (61, 62).

### 4.3 Future development trends

Keywords burst analysis identifies metabolism, EMT, TNBC, treatment resistance, invasion, prognosis, and the TME as persistent hot topics. Analyzing significant literature from the past 5 years, particularly 2024, suggests future trends in breast cancer epigenetics, with a focus on the following directions:

#### 4.3.1 Targeted epigenetic memory clearance for treating drug resistance and recurrence

In this study, keywords such as “epigenetic memory,” “transcription factor,” “cisplatin response,” “dormant breast cancer cell,” and “therapy resistance” were identified in the 2024 clustering, suggesting that this is a potential trend. Studies have shown that genetic changes in gene expression or behavior induced by prior stimuli can be recorded by the organism as epigenetic memory (63). These epigenetic marks facilitate the untimely reactivation of dormant tumor cells, leading to tumor recurrence and treatment resistance (64–66). Bian et al. reported that radiotherapy leads to the epigenetic activation of thrombospondin 1(THBS1), increasing the difficulty of healing cancer wounds, and upon re-injury, the expression of THBS1 increases, affecting wound healing, illustrating the impact of epigenetic memory (67).

As for breast cancer, the HDAC inhibitor trichostatin A helps overcome the resistance of breast cancer cells to tamoxifen (68), which provides reliable evidence for this research direction. A clinical study indicates that treatment in the pre-neoadjuvant window with DNA methyltransferase inhibitor (decitabine, 15 mg/m² × 4 doses over 5 days) and T-cell immune checkpoint inhibition (pembrolizumab, 200 mg, administered 2 weeks apart) combined, is beneficial for the treatment of locally advanced HER2-negative breast cancer (69).

#### 4.3.2 Improving the metabolic and epigenetic interaction network, and treating breast cancer through a synthetic lethal strategy

Currently, researchers generally believe that tumor occurrence results from interactive networks rather than single gene mutations. It has been proven that hypoxia induces epigenetic reprogramming of normal fibroblasts, thereby producing a pro-glycolytic transcriptome similar to that of tumor-associated fibroblasts, leading to the occurrence of breast cancer (22). Combining epigenetic drugs with other antineoplastic agents is a promising treatment strategy for advanced cancers (70, 71). The therapeutic effect of HDAC inhibitors combined with anticancer drugs, such as NSC-3852 combined with olaparib (72), romidepsin and ATR inhibitors (73), is significantly enhanced.

The principle of synthetic lethality, which refers to the loss of viability resulting from the disruption of two genes, which, individually, do not cause lethality This principle is primarily used to treat advanced ovarian and breast cancers associated with BRCA1 or BRCA2 gene mutations (74). Notably, the clinical success of poly (ADP-ribose) polymerase inhibitors (PARPi) has made synthetic lethality an attractive targeted therapeutic strategy; however, its limitation in treatment resistance has also gradually become apparent (75, 76).

In this study, “oxidative phosphorylation,” “synthetic lethality strategies,” and “epigenetic mechanism” were identified, indicating that these may be directions for future research.

#### 4.3.3 Single-cell sequencing tracks the dynamic changes in epigenetic modifications in breast cancer across space and time

Single-cell sequencing technology utilizes optimized next-generation DNA sequencing (NGS) to detect the sequences of individual cells, allowing the acquisition of cell sequence differences in specific microenvironments to facilitate the study of functional differences (77). Scholars have employed single-cell sequencing technology to analyze epigenetic alterations in breast cancer. Fang et al. employed single-cell sequencing analysis and scRNA-seq data to define nine CSs from normal tissues and tamoxifen-treated recurrent tumors. They ultimately demonstrated that BMP7 plays an oncogenic role in tamoxifen-resistant breast cancer cells by modulating MAPK signaling pathways. Cancer-specific enhancers in male breast cancer have also been identified through single-cell sequencing technology (78). In addition, single-cell sequencing technology has been integrated with omics approaches to reanalyze and define the regulatory logic of breast cancer. This integration has identified the most probable cells of origin for subtype-specific breast tumors and implemented linear mixed-effects models to quantify the associations between regulatory elements and gene expression in malignant and normal cells (79).

## 5 Limitations

This bibliometric analysis offers a comprehensive overview of epigenetic research in breast cancer; however, it presents several limitations. First, relying exclusively on the WoSCC database might lead to an incomplete literature compilation, introducing potential biases. Despite this limitation, the WoSCC is recognized as a globally authoritative source, featuring high-impact academic journals and citation indexes, which help maintain the study’s credibility. Second, since there is currently no reliable method for the batch processing of self-citations and for the identification of predatory journals, the research results may be subject to a certain degree of bias. Third, the method’s focus on keyword co-occurrence and citation networks might miss emerging research trends not yet highly cited. To compensate for these deficiencies in recent research evaluations, future researchers may consider integrating journal impact, author influence, and advanced artificial intelligence technologies to develop new comprehensive evaluation models.

## 6 Conclusion

This bibliometric analysis provides a comprehensive overview of the evolution of epigenetic research in breast cancer over the past two decades. This highlights the progression from single-site methylation studies to the systematic exploration of epigenetic pathways and therapies, such as HDAC inhibitors, showcasing the field’s maturation. Future research should focus on targeted epigenetic memory clearance to combat drug resistance and recurrence, enhancement of the metabolic and epigenetic interaction networks, and the application of synthetic lethal strategies in breast cancer treatment. Additionally, interdisciplinary collaborations and the integration of emerging technologies, such as single-cell sequencing, are recommended to facilitate dynamic monitoring of epigenetic changes in drug development.

## Data Availability

The original contributions presented in the study are included in the article/**Supplementary Material**. Further inquiries can be directed to the corresponding author.
