# Supplementary material for: Progress in epigenetic research of breast cancer: a bibliometric analysis since the 2000s
Source: Front Oncol. 2025 Sep 5;15:1619346. doi: 10.3389/fonc.2025.1619346 (PMC12446028; doi:10.3389/fonc.2025.1619346)
Supplement: Supplementary file 1 [file SupplementaryFile1.pdf]

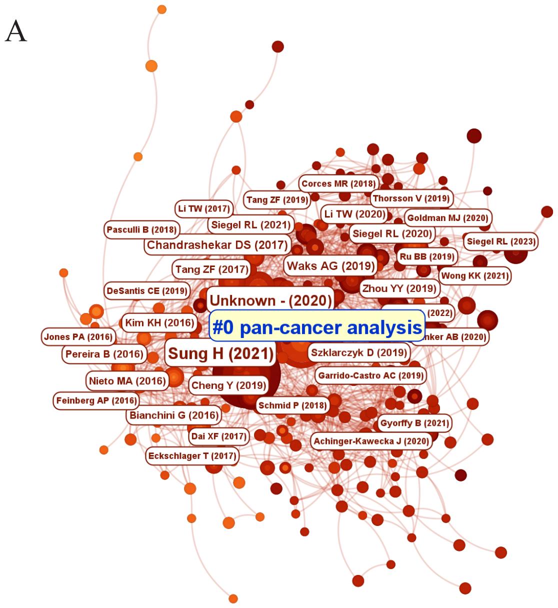

Cluster0 pan-cancer analysis : pan-cancer analysis (1929.08, 1.0E-4); prognostic value (1786.3, 1.0E-4); comprehensive analysis (1696.47, 1.0E-4); chromobox family member (1579.08, 1.0E-4); triple-negative breast cancer (1534.23, 1.0E-4)

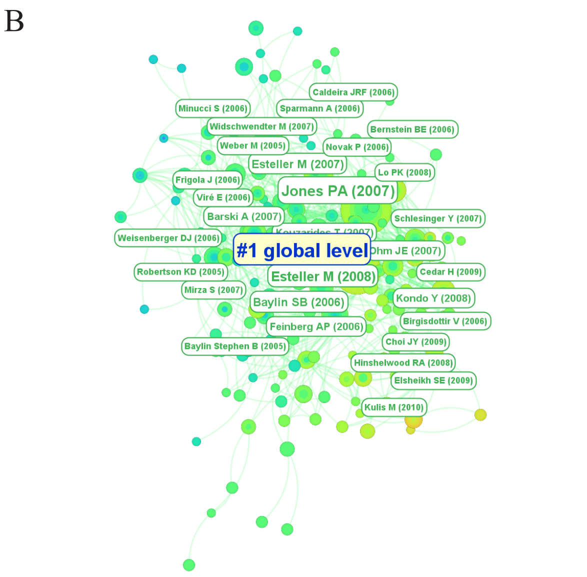

Cluster1 global level : global level (964.18, 1.0E-4); epigenetic signature (956.34, 1.0E-4); triple-negative breast cancer (956.04, 1.0E-4); sporadic breast cancer (906.44, 1.0E-4); new york site (818.95, 1.0E-4)

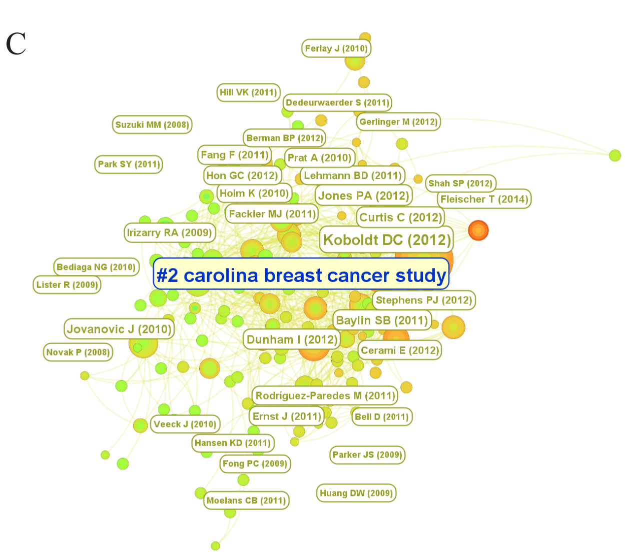

Cluster2 carolina breast cancer study : carolina breast cancer study (856.26, 1.0E-4); dna methylation (794.08, 1.0E-4); invasive breast cancer (595.7, 1.0E-4); distinct pattern (590.41, 1.0E-4); epigenetic determinant (575.64, 1.0E-4)

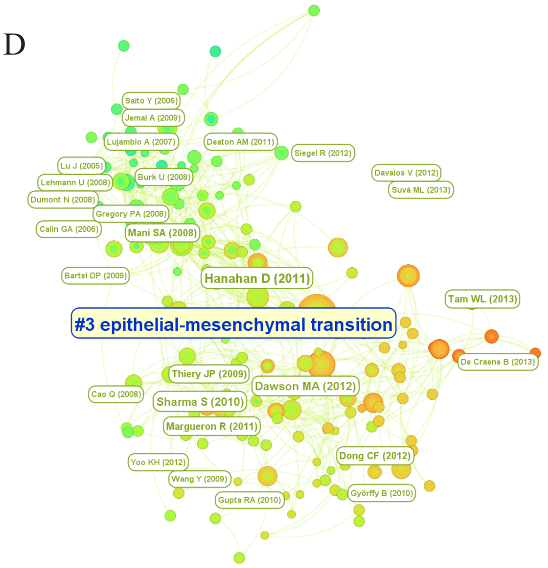

Cluster3 epithelial-mesenchymal transition : epithelial-mesenchymal transition (2681.02, 1.0E-4); to-mesenchymal transition (1595.05, 1.0E-4); epigenetic repression (1519.31, 1.0E-4); epigenetic regulation (1218.35, 1.0E-4); mir-200 family (1052.92, 1.0E-4)

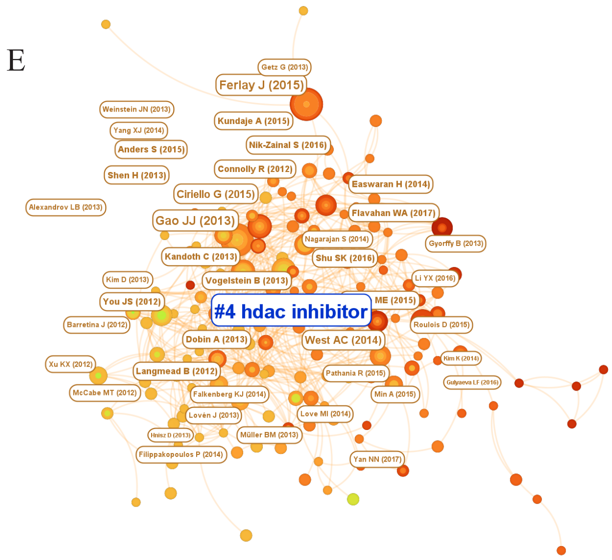

Cluster4 hdac inhibitor : hdac inhibitor (864.12, 1.0E-4); advanced breast cancer (812.31, 1.0E-4); prefrontal cortex (768.41, 1.0E-4); tumorgraft mice (768.41, 1.0E-4); chemotherapeutic agent (728.54, 1.0E-4)

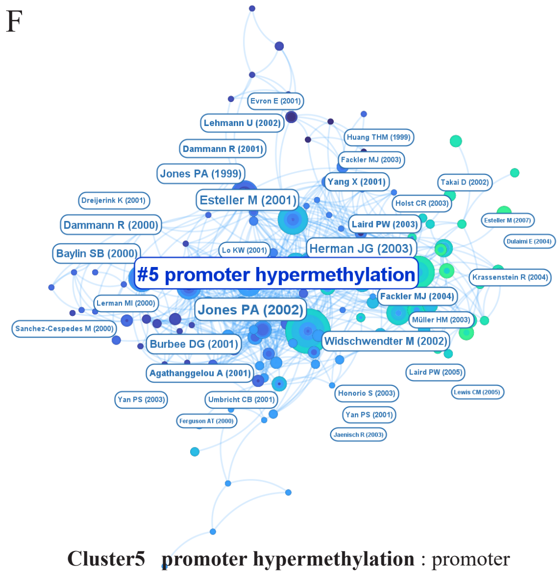

Cluster5 promoter hypermethylation : promoter hypermethylation (1069.45, 1.0E-4); high frequency (817.61, 1.0E-4); human homologue (656.57, 1.0E-4); epigenetic inactivation (534.89, 1.0E-4); cpg island methylation (502.7, 1.0E-4)

G

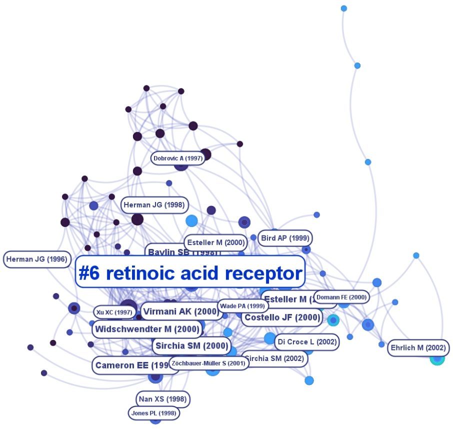

Cluster6 retinoic acid receptor : retinoic acid receptor (1127.22, 1.0E-4); retinoic acid (460.91, 1.0E-4); 2 gene (450.83, 1.0E-4); 5-encoding gene (410.66, 1.0E-4); human prostate cancer (280.2, 1.0E-4)

H

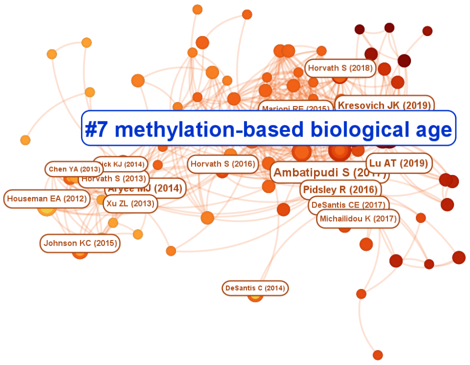

Cluster7 methylation-based biological age : methylation-based biological age (929.63, 1.0E-4); environmental exposure (830.58, 1.0E-4); stochastic epigenetic mutation (698.64, 1.0E-4); epigenetic aging (566.82, 1.0E-4); peripheral blood (516.02, 1.0E-4)

I

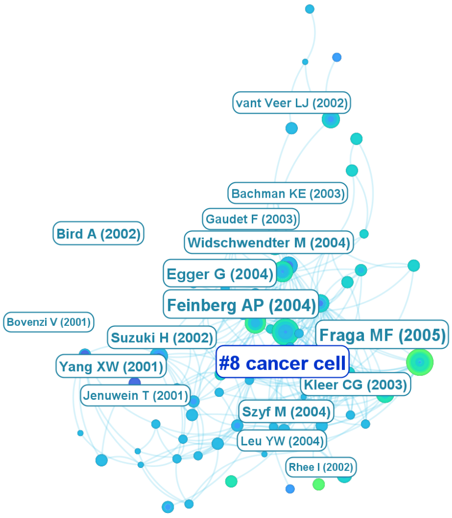

Cluster8 cancer cell : cancer cell (587.66, 1.0E-4); histone deacetylase inhibitor (399.13, 1.0E-4); suv4-20h2 histone methyltransferase (349.31, 1.0E-4); histone h4 (349.31, 1.0E-4); methyl-binding protein (349.31, 1.0E-4)

J

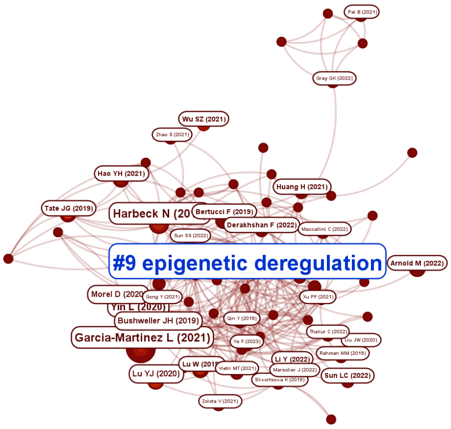

Cluster9 epigenetic deregulation : epigenetic deregulation (387.31, 1.0E-4); breast cancer microenvironment (387.31, 1.0E-4); therapeutic strategies (387.31, 1.0E-4); apoptotic-mediated cell death (378.85, 1.0E-4); apoptotic regulator (378.85, 1.0E-4)

K

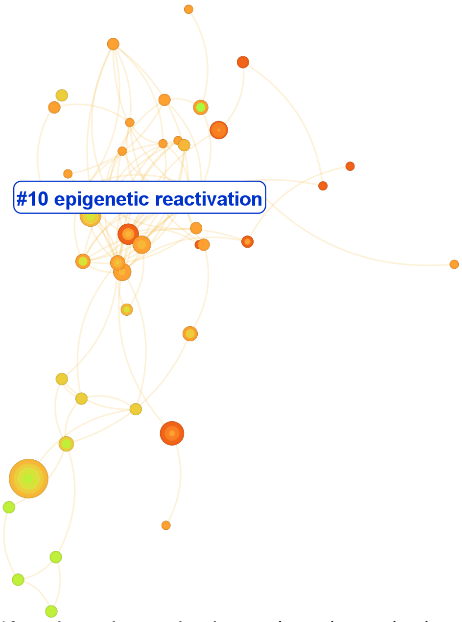

Cluster10 epigenetic reactivation : epigenetic reactivation (713.81, 1.0E-4); negative breast cancer cell (540.06, 1.0E-4); triple negative breast cancer hallmark (431.67, 1.0E-4); complex mediated epigenetic repression (424.45, 1.0E-4); z-ligustilide restore (424.45, 1.0E-4)

L

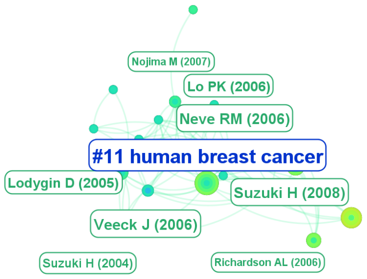

Cluster11 human breast cancer : human breast cancer (368.4, 1.0E-4); novel target (269.69, 1.0E-4); unfavorable prognosis (248.43, 1.0E-4); tumor-specific epigenetic marker (237.79, 1.0E-4); high-frequent alteration (232.16, 1.0E-4)

M

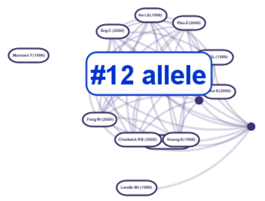

Cluster12 allele : allele (22.81, 1.0E-4); preferential loss (22.81, 1.0E-4); human hepatocellular carcinoma (11.41, 0.001); breast cancer (0.41, 1.0); triple-negative breast cancer (0.09, 1.0)

N

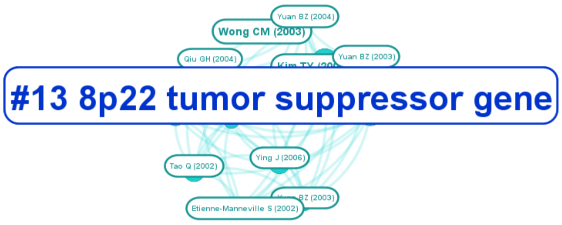

Cluster13 8p22 tumor suppressor gene : 8p22 tumor suppressor gene (74.18, 1.0E-4); tumor-specific methylation (55.49, 1.0E-4); other type (55.49, 1.0E-4); sporadic nasopharyngeal esophageal (36.9, 1.0E-4); tumor cell colony formation (36.9, 1.0E-4)

O

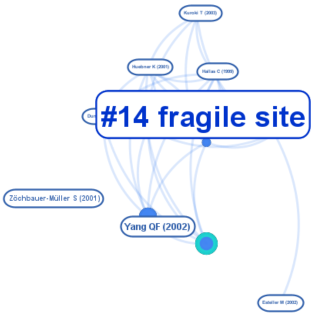

Cluster14 fragile site : fragile site (56.39, 1.0E-4); common chromosome (56.39, 1.0E-4); hematopoietic malignancies (56.39, 1.0E-4); burkitts lymphoma (37.49, 1.0E-4); frequent silencing (37.49, 1.0E-4)

P

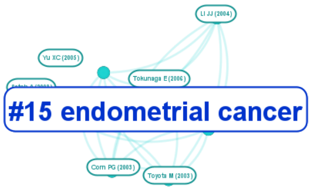

Cluster15 endometrial cancer : endometrial cancer (21.63, 1.0E-4); relationship (21.63, 1.0E-4); taxane (21.63, 1.0E-4); sensitivity (21.63, 1.0E-4); aberrant dna hypermethylation (12.23, 0.001)

Supplementary Fig. S 1 Detailed information on the most significant clusters within the co-citation network of references, ranked by citation bursts, is provided for the period 2000–2024.

For each cluster, we listed the top 5 keywords, and labeled the cluster with the most cited keyword (generated by comparing the likelihood ratio of keywords). These keywords are highly predictive of the overall topic of a cluster.

A

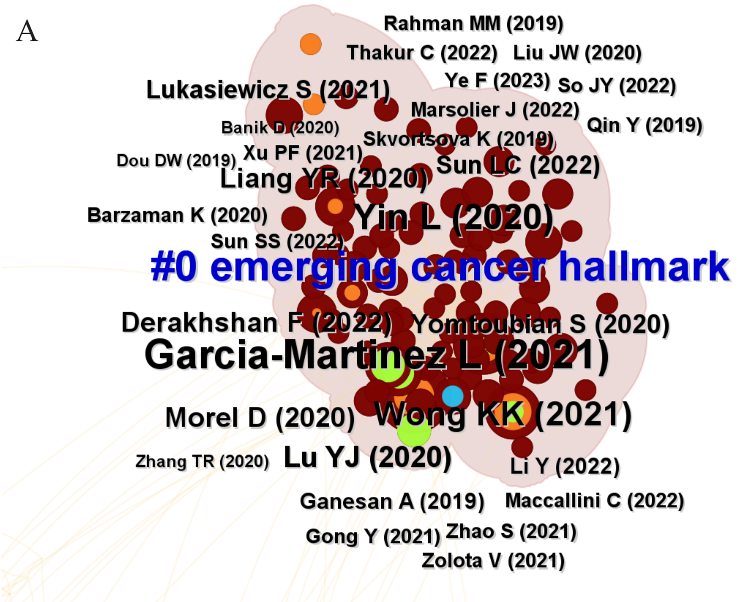

Cluster0 emerging cancer hallmark: emerging cancer hallmark (175.96, 1.0E-4); apoptotic-mediated cell death (170.77, 1.0E-4); molecular determinant (165.59, 1.0E-4); mechanistic insight (165.59, 1.0E-4); epigenetic activation (165.59, 1.0E-4)

B

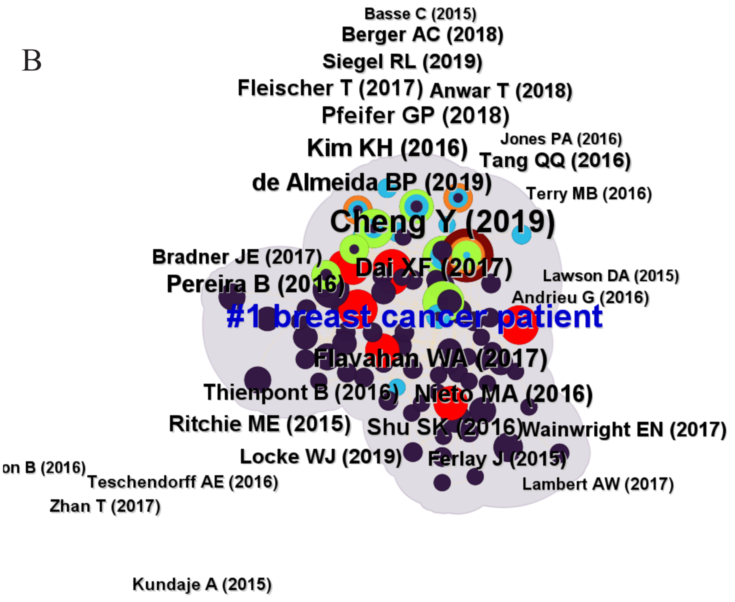

Cluster1 breast cancer patient: breast cancer patient (259.58, 1.0E-4); clinical risk assessment (228.71, 1.0E-4); stemness-related gene panel (200.05, 1.0E-4); bet inhibition (200.05, 1.0E-4); single-cell epigenomics (195.96, 1.0E-4)

C

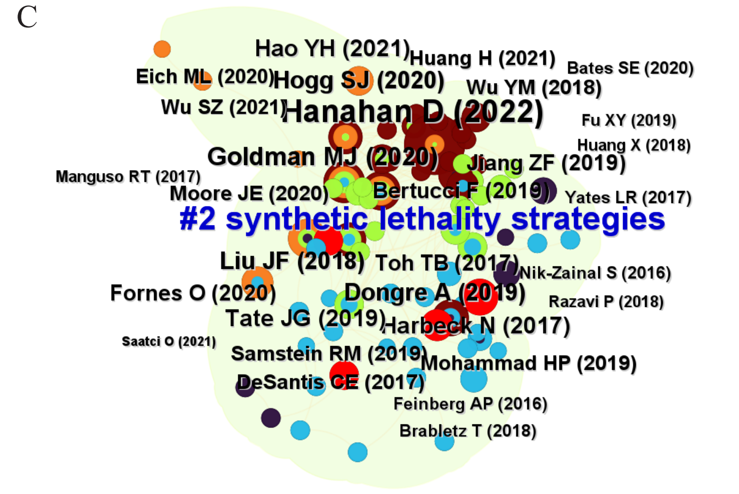

Cluster2 synthetic lethality strategies: synthetic lethality strategies (175.88, 1.0E-4); dna damage responses (175.88, 1.0E-4); targeting histone (175.88, 1.0E-4); epigenetic adaptation route (171.78, 1.0E-4); causes cell death (167.69, 1.0E-4)

D

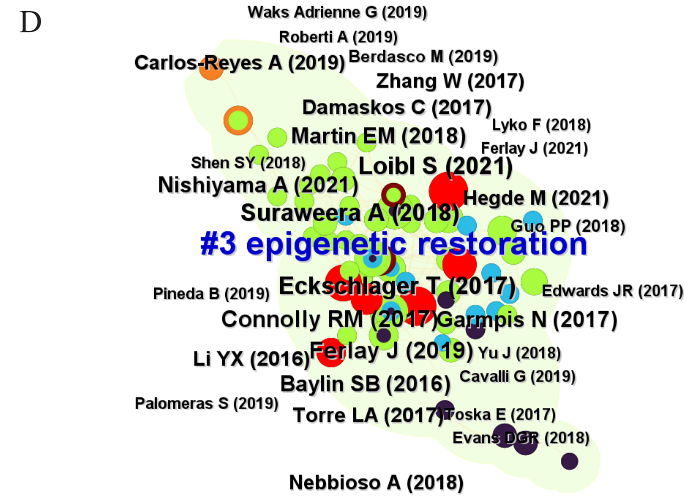

Cluster3 epigenetic restoration: epigenetic restoration (199.52, 1.0E-4); therapeutic application (189.76, 1.0E-4); breast cancer therapy (189.76, 1.0E-4); current paradigm (184.88, 1.0E-4); epigenetic anticancer therapeutics (184.88, 1.0E-4)

E

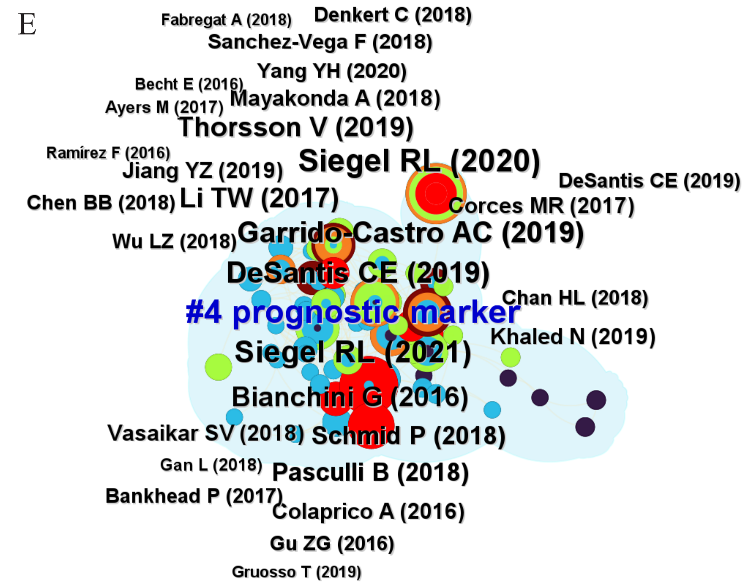

Cluster4 prognostic marker: prognostic marker (337.62, 1.0E-4); multi-omics analysis (306.38, 1.0E-4); alternative splicing (220.45, 1.0E-4); myc suppresses (187.85, 1.0E-4); non-inflamed tumor microenvironment (183.91, 1.0E-4)

F

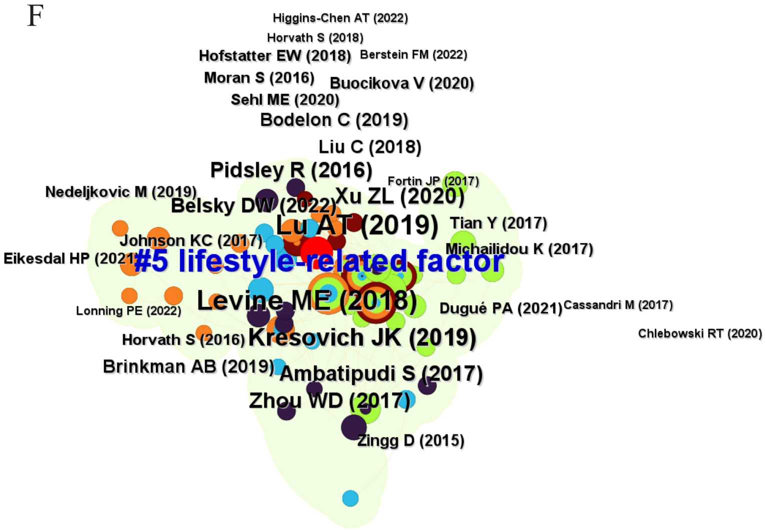

Cluster5 lifestyle-related factor: lifestyle-related factor (246.76, 1.0E-4); methylation-based marker (233.43, 1.0E-4); epigenetic age (193.56, 1.0E-4); prospective studies (174.76, 1.0E-4); epigenetic aging (174.59, 1.0E-4)

G

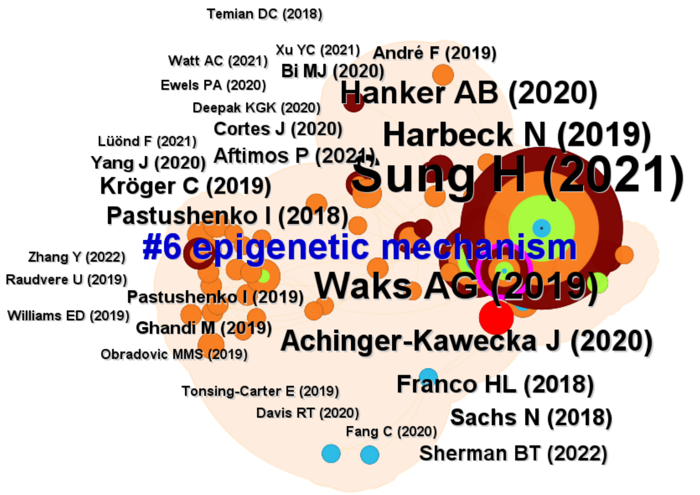

Cluster6 epigenetic mechanism: epigenetic mechanism (263.54, 1.0E-4); tumor progression (224.83, 1.0E-4); mll3 loss drives metastasis (141.87, 1.0E-4); hybrid epithelial-mesenchymal transition state (141.87, 1.0E-4); clonal propagation (139.06, 1.0E-4)

H

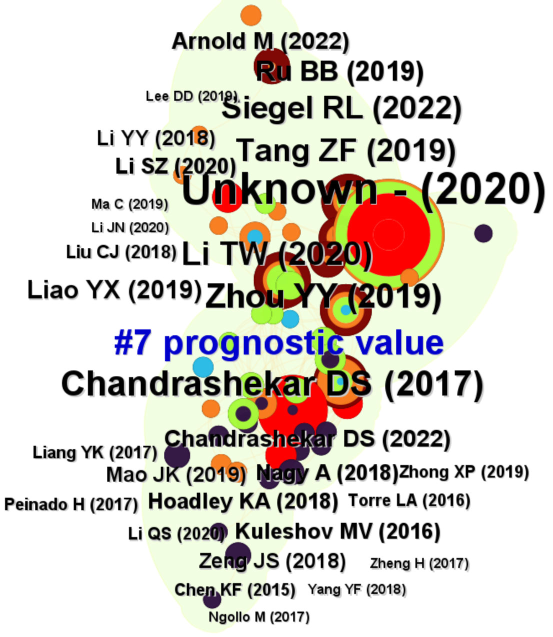

Cluster7 prognostic value: prognostic value (549.58, 1.0E-4); chromobox family member (426.77, 1.0E-4); gastric cancer (393.32, 1.0E-4); triple-negative breast cancer (237.76, 1.0E-4); receptor-negative mammary cancer (205.6, 1.0E-4)

I

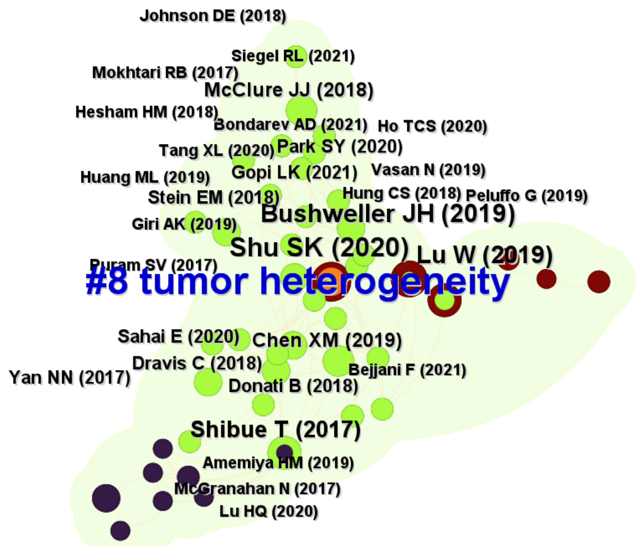

Cluster8 tumor heterogeneity: tumor heterogeneity (163.92, 1.0E-4); histone modification (157.09, 1.0E-4); epigenomic interplay (155.66, 1.0E-4); adjunct therapy (155.66, 1.0E-4); ap-1 oncoprotein (149.62, 1.0E-4)

J

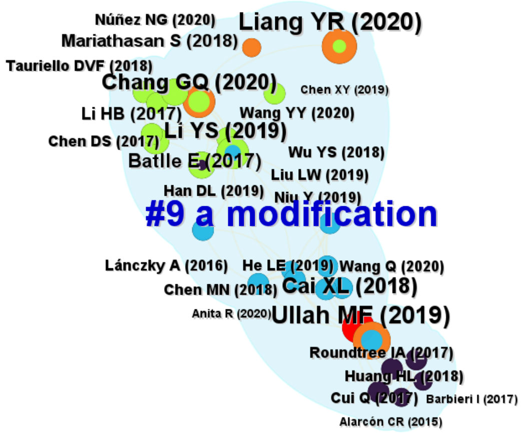

Cluster9 a modification: a modification (115.9, 1.0E-4); 6-methyladenosine enzyme (105.76, 1.0E-4); m6a writers eraser (95.65, 1.0E-4); invasive carcinoma (85.57, 1.0E-4); a pathway regulator (85.57, 1.0E-4)

K

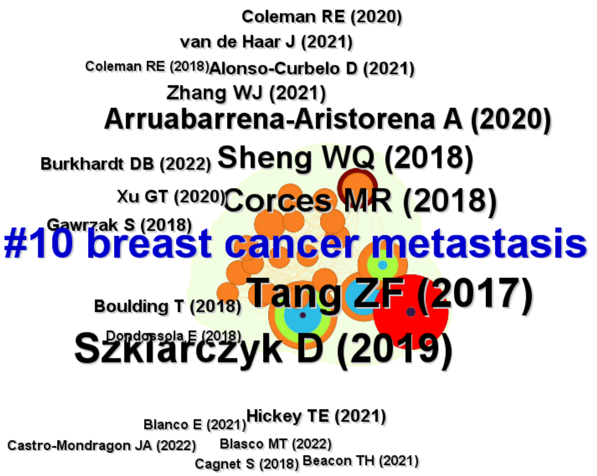

Cluster10 breast cancer metastasis: breast cancer metastasis (176.93, 1.0E-4); amplification licenses (123.68, 1.0E-4); bone metastasis (116.26, 1.0E-4); specific targeted nanomedicine (116.26, 1.0E-4); enhanced treatment (116.26, 1.0E-4)

L

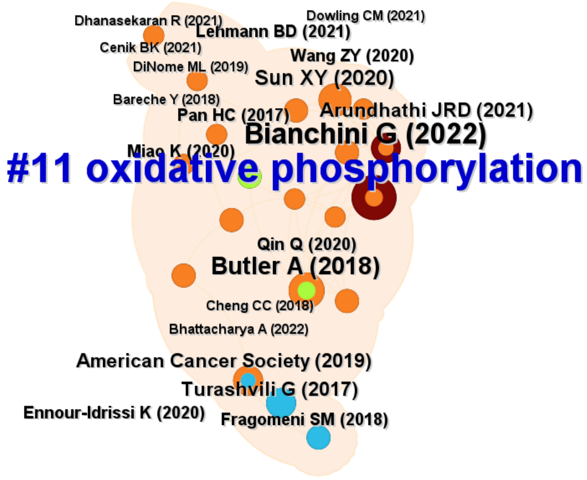

Cluster11 oxidative phosphorylation: oxidative phosphorylation (107.15, 1.0E-4); triple-negative breast cancer stem cell (107.15, 1.0E-4); aerobic glycolysis (107.15, 1.0E-4); muc1-c integrate (107.15, 1.0E-4); dual inhibitor zmf-23 (97.37, 1.0E-4)

M

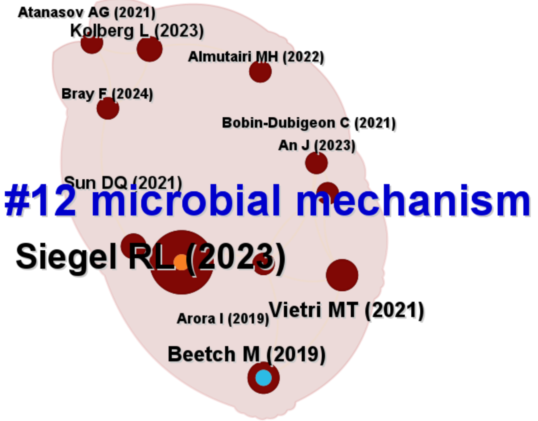

Cluster12 microbial mechanism: microbial mechanism (81.72, 1.0E-4); combinatorial approach (81.72, 1.0E-4); receptor-negative breast cancer (81.72, 1.0E-4); a-rich extract (81.72, 1.0E-4); using sulforaphane- (81.72, 1.0E-4)

N

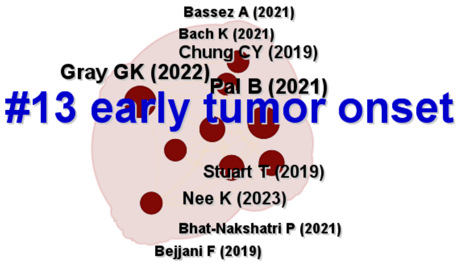

Cluster13 early tumor onset: early tumor onset (40.12, 1.0E-4); mouse model (40.12, 1.0E-4); hereditary breast cancer (40.12, 1.0E-4); epigenetic alteration (40.12, 1.0E-4); single-cell analyses (25.82, 1.0E-4)

O

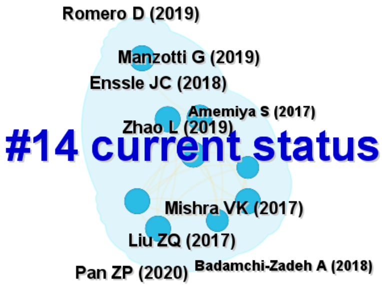

Cluster14 current status: current status (81.14, 1.0E-4); hdac inhibitor (48.04, 1.0E-4); histone acetylation modulator gene (15.82, 1.0E-4); triple-negative breast cancer (0.55, 0.5); prognostic value (0.31, 1.0)

Supplementary Fig. S 2 Detailed information on the most significant clusters within the co-citation network of references, ranked by citation bursts, is provided for the period 2000–2024.

For each cluster, we listed the top 5 keywords, and labeled the cluster with the most cited keyword (generated by comparing the likelihood ratio of keywords). These keywords are highly predictive of the overall topic of a cluster.
